# Network pharmacological prediction of the mechanism of action of Shen-Zhu-Lian-Bai Decoction in the treatment of ulcerative colitis

**DOI:** 10.1038/s41598-024-64683-4

**Published:** 2024-06-20

**Authors:** Li Zhu, Jinghua Liang

**Affiliations:** 1 Anorectal Surgery, Shenzhen TCM Anorectal Hospital, Shenzhen, Guangdong China; 2 Anorectal Surgery, Meizhou People′s Hospital, Meizhou, Guangdong China

**Keywords:** Ulcerative colitis, Shen-Zhu-Lian-Bai decoction (SZLBD), Network pharmacological, Molecular docking, Cell experiments, Clinical pharmacology, Pharmacogenetics

## Abstract

The incidence of ulcerative colitis (UC) is on the rise globally. Shen-Zhu-Lian-Bai decoction (SZLBD) can relieve the clinical symptoms of UC. This study aimed to investigate the underlying molecular mechanism of SZLBD in the treatment of UC. The key treatment targets of SZLBD for UC were obtained based on the online database, and combined with the STRING database and Cytoscape 3.7.2 software, PPI network was constructed and visualized. The GEO database was utilized to validate the expression levels of core targets in UC. Metascape database GO functional annotation and KEGG pathway enrichment analysis. Molecular docking technology was used to verify the docking of core compounds with key targets. RT-qPCR and Western Blot were used to detect the expression of key targets in HCoEpiC cells for verification. After screening, 67 targets shared by SZLBD and UC were obtained. It is predicted that *IL-6*, *IL-1B*, and *AKT1* might be the key targets of SZLBD in the treatment of UC. Quercetin was the main active ingredient. GEO results showed that the expression levels of IL-6, IL-1B and AKT1 were higher in the UC group compared to the control group. GO and KEGG analyses showed that these targets were related to apoptosis and inflammation. The results of molecular docking demonstrated that the *AKT1* gene, a key target of quercetin, had the highest affinity of -9.2 kcal/mol. Cell experiments found that quercetin could affect the expression of *IL-6*, *IL-1B,* and *AKT1*. This study preliminarily explored and verified the mechanism of action of SZLBD in the treatment of UC, which provides a theoretical basis for subsequent in vivo mechanism studies.

## Introduction

Ulcerative colitis (UC) is a chronic inflammatory disease of the colon that typically presents with abdominal pain, diarrhea, and blood in the stool^1^. It is characterized by recurrence and remission of mucosal inflammation that begins in the rectum and extends near the colon. Endoscopic biopsy is the only way to diagnose UC^2^. Currently, the incidence of UC is on the rise globally^2^. Although the exact pathogenesis of UC is unknown, several factors, such as defects in the colonic epithelium, the mucus barrier, and the epithelial barrier^3^ have been identified to affect UC development. Medications for UC include 5-aminosalicylic acid drugs (5-ASA), steroids, and immunosuppressant. Although Western medicine can achieve certain an effect, the effect is not ideal^4^.

Many herbs have shown significant advantages in treating UC^5^. The commonly used traditional Chinese medicines (TCM) formulas for the treatment of UC include Ge-Gen-Qin-Lian decoction (GGQLD)^6^ and Shen-Ling-Bai-Zhu-San (SLBZS)^7^. Based on these two formulas, we combined clinical conditions to develop the TCM formula Shen-Zhu-Lian-Bai decoction (SZLBD). SZLBD is composed of *Coptis chinensis* Franch. (Huanglian), *Phellodendron chinense* Schneid. (Huangbai), *Atractylodes macrocephala* Koidz. (Baizhu), *Portulacae oleracea* L. (Machixian), *Citrus reticulata* Blanco (Chenpi), *Codonopsis pilosula* (Franch.) Nannf. (Dangshen), *Paeonia lactiflora* Pall. (Baishao). Huanglian and its active ingredient berberine have been proved to treat UC^8^. The ingredient of Huangbai can treat UC^9^. Baizhu Shaoyao San (BSS) composed of Baizhu, Baishao and Chenpi, so on, which can be used to treat UC^10^. Dangshen combined with astragalus polysaccharide (APS) can improve colitis in mice^11^. The above research indicates that the components of SZLBD play a role in treating UC. Therefore, we believe that SZLBD may have a good therapeutic effect on UC, but its related mechanism of action is still unclear.

**Figure 1:**
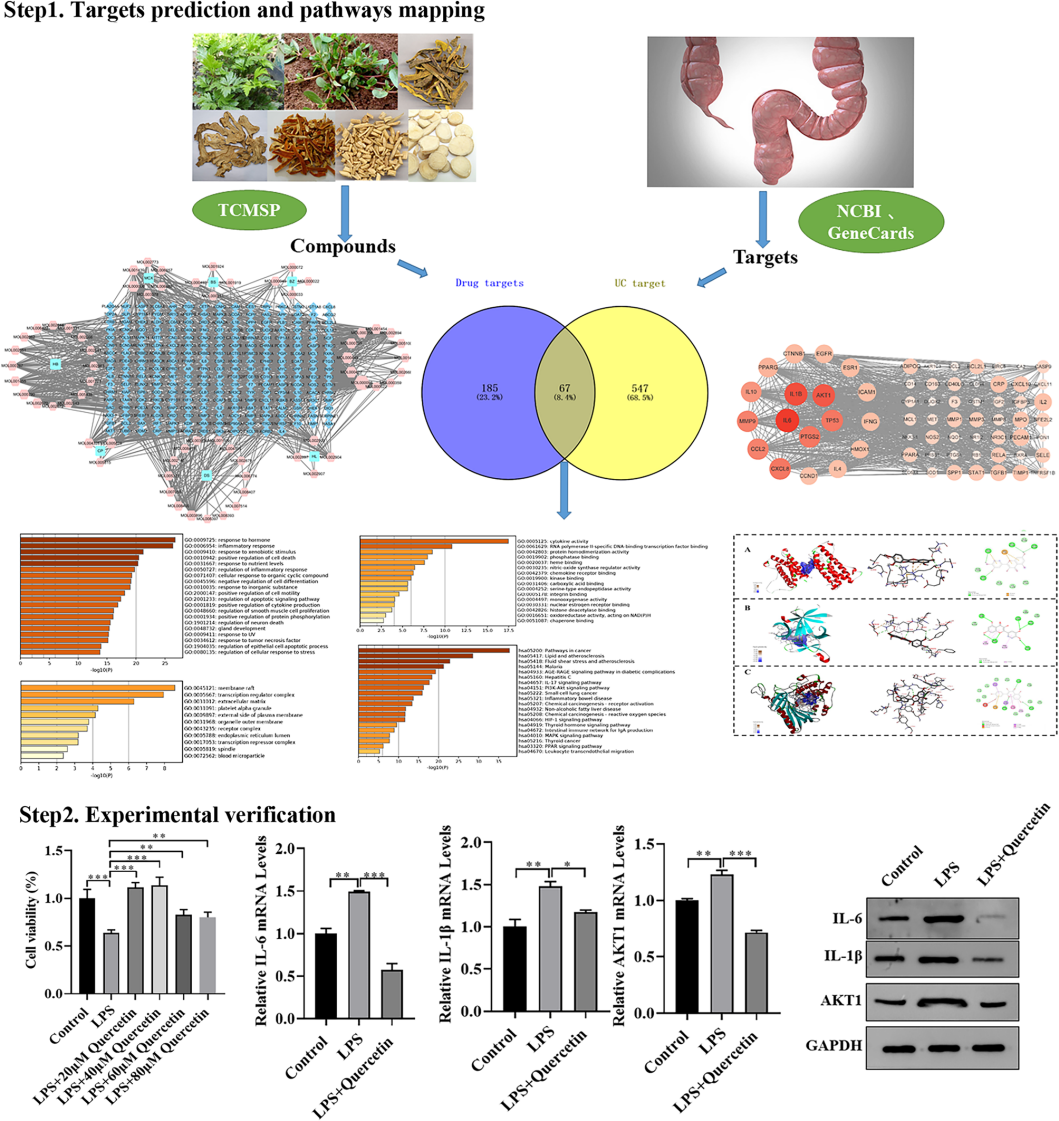
Flow chart of SZLBD against UC.

In this study, TCM formula SZLBD was used as the research object, and network pharmacology and molecular docking methods were applied to predict the effective compounds, key targets and signaling pathways of SZLBD in the treatment of UC, which were verified by cell experiments. The specific flow chart is shown in Fig. 1.

## Materials and methods

### Screening of active compounds in SZLBD

The active compounds of 7 TCM in SZLBD were searched in the Traditional Chinese Medicine Systems Pharmacology (TCMSP) database (http://tcmspw.com/), and each compounds was screened according to the criteria of by the oral bioavailability (OB) ≥ 30% and drug likeness (DL) ≥ 0.18.

### Screening of common targets of SZLBD and UC

The active compounds of SZLBD and their target information were retrieved from the TCMSP database, and the targets were converted into the corresponding human gene names using the STRING database (https://string-db.org/). UC-associated targets were obtained from the GeneCards database (https://www.genecards.org/) and the NCBI database by entering the keyword “ulcerative colitis”. All targets were limited to Homo sapiens. Next, the common targets of GeneCards and NCBI databases were retained as UC-related targets. Finally, the intersection targets of SZLBD-related targets and UC-related targets were considered as potential therapeutic target for SZLBD in the treatment of UC.

### Protein–protein interaction (PPI) networks construction

The common targets of SZLBD and UC were entered into the STRING database (https://string-db.org/) for PPI network construction. The nodes and edges of the network represent proteins and protein–protein associations, respectively. PPI networks were visualized using Cytoscape 3.7.2 software, and topological features were analyzed to screen core targets.

### GEO verification

We validated the expression levels of these core targets in UC using an external dataset from the Gene Expression Omnibus (GEO). We utilized the "limma" package in R language to obtain the expression levels of core genes in both the normal and UC groups. We then generated box plots using the Sangerbox online platform (http://sangerbox.com/home.html).

### GO functional annotation and KEGG signaling pathway enrichment analysis

In order to understand the biological functions and related signaling pathways involved in the common targets of SZLBD and UC, GO functional annotation (including biological process (BP), cell ingredient (CC) and molecular function (MF)) and KEGG signaling pathway enrichment analysis were performed for the common targets using Metascape database (https://metascape.org/). In the end, there are only *p*-values < 0.01, count > 3, enrichment factor (enrichment factor is the ratio between observed counts and counts expected by chance) > 1.5 is considered a significant enrichment result^12,13^.

### Molecular docking

The molecular structure of the core active compounds were downloaded from the PubChem database (https://pubchem.ncbi.nlm.nih.gov/). The protein crystal structure of the key targets were obtained from the RCSB PDB database (PDB, https://www.rcsb.org/). The structure of compounds and proteins were processed by AutoDockTools (version 1.5.6). Optimal combinations of docking score were visualized by PyMOL.

## Experimental verification

### Cell culture and quercetin administration

Human colonic epithelial cells (HCoEpiC) were purchased from Saibaikang Company (Shanghai, China). HCoEpiC cells were cultured at 37 °C under 5% CO_2_-humidified air in DMEM supplemented with 10% fetal bovine serum (FBS), 100 U/mL penicillin and 100 μg/mL streptomycin^14^.

### Screening of LPS concentration

HCoEpiC was made into 5 × 10^4^ cells/mL suspension, inoculated in culture plates or culture flasks, and cultured in a 37 °C incubator with 5% CO_2_ for 24 h. Cells were treated with different concentrations of LPS (5, 10, 15 μg/mL). After 24 h, cell viability was assessed using the CCK-8 assay by measuring absorbance in each well to calculate cell survival rates.

### Quercetin cytotoxicity test

Quercetin was purchased from MCE Company. The cells were randomly divided into 7 groups, i.e., the normal group, the model group, and the quercetin-added group (0, 20, 40, 60, and 80 μM). Except the normal group, all groups were given LPS with a final concentration of 10 μg/mL to create an inflammatory injury model. In addition to the normal group and the model group, each group was added with the corresponding test drug and incubated at 37 °C with 5% CO_2_ for 24 h. Cell viability was assessed using the CCK-8 assay by measuring absorbance in each well to calculate cell survival rates.

### Real-time quantitative PCR (RT-qPCR)

Total cellular RNA was extracted using TRIzol reagent (Invitrogen, Carlsbad, CA, USA) according to the manufacturer’s instructions. Subsequently, cDNA was synthesized using a PrimeScript RT reagent kit (Takara Biotechnology, Dalian, China). Real-time PCR was then performed using an ABI 7500 Sequence Detection System (Applied Biosystems, Foster City, CA, USA). The primer sequences are shown in Table 1.

**Table 1:** Primer Sequence.

Gene	Primer sequence
AKT1-F	TGGACTACCTGCACTCGGAGAA
AKT1-R	GTGCCGCAAAAGGCTTTCATGG
IL-6-F	CTCCTTCTCCACAAGCGCC
IL-6-R	GATGCCGTCGAGGATGTACC
IL-1B-F	GCCAGTGAAATGATGGCTTATT
IL-1B-R	AGGAGCACTTCATCTGTTTAGG
GAPDH-F	GGAGCGAGATCCCTCCAAAAT
GAPDH-R	GGCTGTTGTCATACTTCTCATGG

*F* forward, *R* reverse.

### Western blot analysis

The cells were lysed using RIPA lysis buffer after treatment. After centrifugation at 4 °C and 3000 rpm for 10 min, the supernatant was collected. Subsequently, protein lysates were separated and electrophoresed on PVDF membranes using 10% SDS-PAGE. The PVDF membranes were then incubated in 5% skim milk TBST solution for 2 h, followed by washing with TBST solution without skim milk three times for 10 min each. Afterwards, the membranes were incubated overnight at 4 °C with IL-6, IL-1B, and AKT1 rabbit polyclonal antibodies (sourced respectively from ZEN BIO, proteintech, and ZEN BIO). The membranes were moistened with TBST solution three times for 10 min each, followed by incubation at room temperature for 1.5 h in HRP-labeled goat anti-rabbit IgG solution (dilution ratio: 1/20,000). Finally, the membranes were washed with TBST solution three times for 10 min each, and then tested using a Tanon chemiluminescence imager. The images were quantitatively analyzed using ImageJ software.

## Results

### Screening results of active compounds in SZLBD

**Figure 2:**
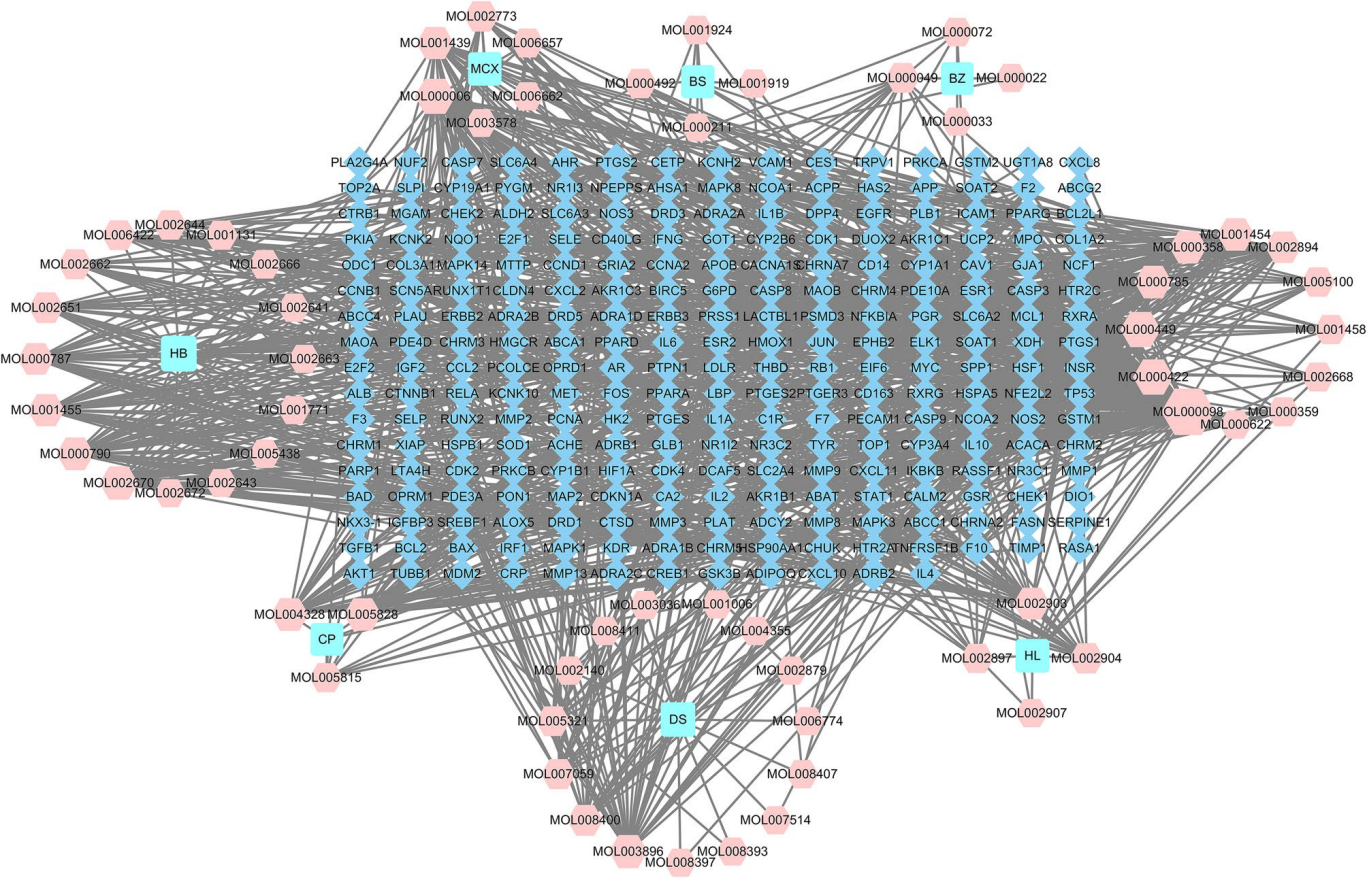
Key active compounds of SZLBD-target network. The teal represents traditional Chinese medicines (HL, Huanglian; CP, Chenpi; BZ, Baizhu; HB, Huangbai; MCX, Machixian; BS, Baishao; DS, Dangshen), the pink represents active ingredients, and the blue represents the targets of SZLBD.

A total of 90 active compounds of SZLBD were obtained based on the TCMSP database and the screening criteria of OB ≥ 30% and DL ≥ 0.18 (Table 2). Combined with the TCMSP database, 185 SZLBD related targets were obtained, and the ingredients—compounds -target network was shown in Fig. 2. As a result, the main active compounds of SZLBD included quercetin and kaempferol, among others, each of which regulated multiple targets.

**Table 2:** Information for Potential active ingredients of SZLBD.

Mol ID	Molecule name	Herb Pinyin	OB (%)	DL
MOL000622	Magnograndiolide	Huanglian, Huangbai	63.709	0.188
MOL008647	Moupinamide	Huanglian	86.712	0.265
MOL000098	Quercetin	Huanglian, Huangbai, Machixian	46.433	0.275
MOL000785	Palmatine	Huanglian, Huangbai	64.601	0.645
MOL000762	Palmidin A	Huanglian, Huangbai	35.358	0.650
MOL002894	Berberrubine	Huanglian, Huangbai	35.736	0.727
MOL013352	Obacunone	Huanglian, Huangbai	43.286	0.767
MOL002903	(R)-Canadine	Huanglian	55.367	0.775
MOL002907	Corchoroside A_qt	Huanglian	104.954	0.776
MOL002897	Epiberberine	Huanglian	43.092	0.776
MOL001454	Berberine	Huanglian, Huangbai	36.861	0.777
MOL002904	Berlambine	Huanglian	36.681	0.816
MOL001458	Coptisine	Huanglian, Huangbai	30.672	0.856
MOL002668	Worenine	Huanglian, Huangbai	45.833	0.866
MOL002659	Kihadanin A	Huangbai	31.605	0.702
MOL002671	Candletoxin A	Huangbai	31.811	0.688
MOL002666	Chelerythrine	Huangbai	34.184	0.780
MOL002636	Kihadalactone A	Huangbai	34.209	0.817
MOL006413	Phellochin	Huangbai	35.412	0.815
MOL002670	Cavidine	Huangbai	35.642	0.805
MOL000790	Isocorypalmine	Huangbai	35.768	0.592
MOL002641	Phellavin_qt	Huangbai	35.860	0.442
MOL002673	Hispidone	Huangbai	36.181	0.830
MOL002656	Dihydroniloticin	Huangbai	36.426	0.815
MOL006392	Dihydroniloticin	Huangbai	36.426	0.815
MOL000358	Beta-sitosterol	Huangbai, Machixian, Baishao	36.914	0.751
MOL001771	Poriferast-5-en-3beta-ol	Huangbai	36.914	0.750
MOL002643	Delta 7-stigmastenol	Huangbai	37.423	0.751
MOL005438	Campesterol	Huangbai	37.577	0.715
MOL002672	Hericenone H	Huangbai	38.997	0.634
MOL002663	Skimmianin	Huangbai	40.137	0.196
MOL002644	Phellopterin	Huangbai	40.186	0.279
MOL002662	Rutaecarpine	Huangbai	40.300	0.598
MOL006401	Melianone	Huangbai	40.529	0.778
MOL002660	Niloticin	Huangbai	41.414	0.818
MOL002651	Dehydrotanshinone II A	Huangbai	43.762	0.400
MOL000449	Stigmasterol	Huangbai, Dangshen	43.830	0.757
MOL006422	Thalifendine	Huangbai	44.411	0.726
MOL001455	(S)-Canadine	Huangbai	53.834	0.775
MOL002652	Delta7-Dehydrosophoramine	Huangbai	54.450	0.253
MOL001131	Phellamurin_qt	Huangbai	56.597	0.393
MOL000787	Fumarine	Huangbai	59.263	0.827
MOL000020	12-senecioyl-2E,8E,10E-atractylentriol	Baizhu	62.396	0.223
MOL000021	14-acetyl-12-senecioyl-2E,8E,10E-atractylentriol	Baizhu	60.313	0.305
MOL000022	14-acetyl-12-senecioyl-2E,8Z,10E-atractylentriol	Baizhu	63.371	0.300
MOL000028	α-Amyrin	Baizhu	39.512	0.763
MOL000033	(3S,8S,9S,10R,13R,14S,17R)-10,13-dimethyl-17-[(2R,5S)-5-propan-2-yloctan-2-yl]-2,3,4,7,8,9,11,12,14,15,16,17-dodecahydro-1H-cyclopenta[a]phenanthren-3-ol	Baizhu	36.228	0.783
MOL000049	3β-acetoxyatractylone	Baizhu	54.067	0.219
MOL000072	8β-ethoxy atractylenolide III	Baizhu	35.951	0.211
MOL001439	Arachidonic acid	Machixian	45.573	0.204
MOL003578	Cycloartenol	Machixian	38.686	0.781
MOL002773	Beta-carotene	Machixian	37.184	0.584
MOL000422	Kaempferol	Machixian, Baishao	41.882	0.241
MOL005100	5,7-dihydroxy-2-(3-hydroxy-4-methoxyphenyl)chroman-4-one	Machixian, Chenpi	47.736	0.272
MOL000006	Luteolin	Machixian, Dangshen	36.163	0.246
MOL006657	Isobetanidin	Machixian	59.731	0.521
MOL006662	Isobetanin_qt	Machixian	30.162	0.521
MOL000359	Sitosterol	Chenpi, Baishao	36.914	0.751
MOL004328	Naringenin	Chenpi	59.294	0.211
MOL005815	Citromitin	Chenpi	86.904	0.514
MOL005828	Nobiletin	Chenpi	61.669	0.517
MOL001006	Poriferasta-7,22E-dien-3beta-ol	Dangshen	42.979	0.756
MOL002140	Perlolyrine	Dangshen	65.948	0.275
MOL002879	Diop	Dangshen	43.593	0.392
MOL003036	ZINC03978781	Dangshen	43.830	0.756
MOL003896	7-Methoxy-2-methyl isoflavone	Dangshen	42.565	0.199
MOL004355	Spinasterol	Dangshen	42.979	0.755
MOL004492	Chrysanthemaxanthin	Dangshen	38.724	0.584
MOL005321	Frutinone A	Dangshen	65.904	0.342
MOL006554	Taraxerol	Dangshen	38.403	0.767
MOL006774	Stigmast-7-enol	Dangshen	37.423	0.751
MOL007059	3-beta-Hydroxymethyllenetanshiquinone	Dangshen	32.161	0.409
MOL007514	Methyl icosa-11,14-dienoate	Dangshen	39.667	0.229
MOL008391	5alpha-Stigmastan-3,6-dione	Dangshen	33.115	0.790
MOL008393	7-(beta-Xylosyl)cephalomannine_qt	Dangshen	38.327	0.286
MOL008397	Daturilin	Dangshen	50.365	0.768
MOL008400	Glycitein	Dangshen	50.479	0.238
MOL008406	Spinoside A	Dangshen	39.967	0.403
MOL008407	(8S,9S,10R,13R,14S,17R)-17-[(E,2R,5S)-5-ethyl-6-methylhept-3-en-2-yl]-10,13-dimethyl-1,2,4,7,8,9,11,12,14,15,16,17-dodecahydrocyclopenta[a]phenanthren-3-one	Dangshen	45.405	0.762
MOL008411	11-Hydroxyrankinidine	Dangshen	40.003	0.662
MOL001910	11alpha,12alpha-epoxy-3beta-23-dihydroxy-30-norolean-20-en-28,12beta-olide	Baishao	64.774	0.376
MOL001918	Paeoniflorgenone	Baishao	87.593	0.367
MOL001919	(3S,5R,8R,9R,10S,14S)-3,17-dihydroxy-4,4,8,10,14-pentamethyl-2,3,5,6,7,9-hexahydro-1H-cyclopenta[a]phenanthrene-15,16-dione	Baishao	43.556	0.533
MOL001921	Lactiflorin	Baishao	49.121	0.797
MOL001924	Paeoniflorin	Baishao	53.870	0.787
MOL001925	Paeoniflorin_qt	Baishao	68.176	0.395
MOL001928	Albiflorin_qt	Baishao	66.641	0.326
MOL001930	Benzoyl paeoniflorin	Baishao	31.274	0.746
MOL000211	Mairin	Baishao	55.377	0.776
MOL000492	( +)-catechin	Baishao	54.826	0.242

*SZLBD* Shen-Zhu-Lian-Bai decoction.

### Common targets of SZLBD and UC

**Figure 3:**
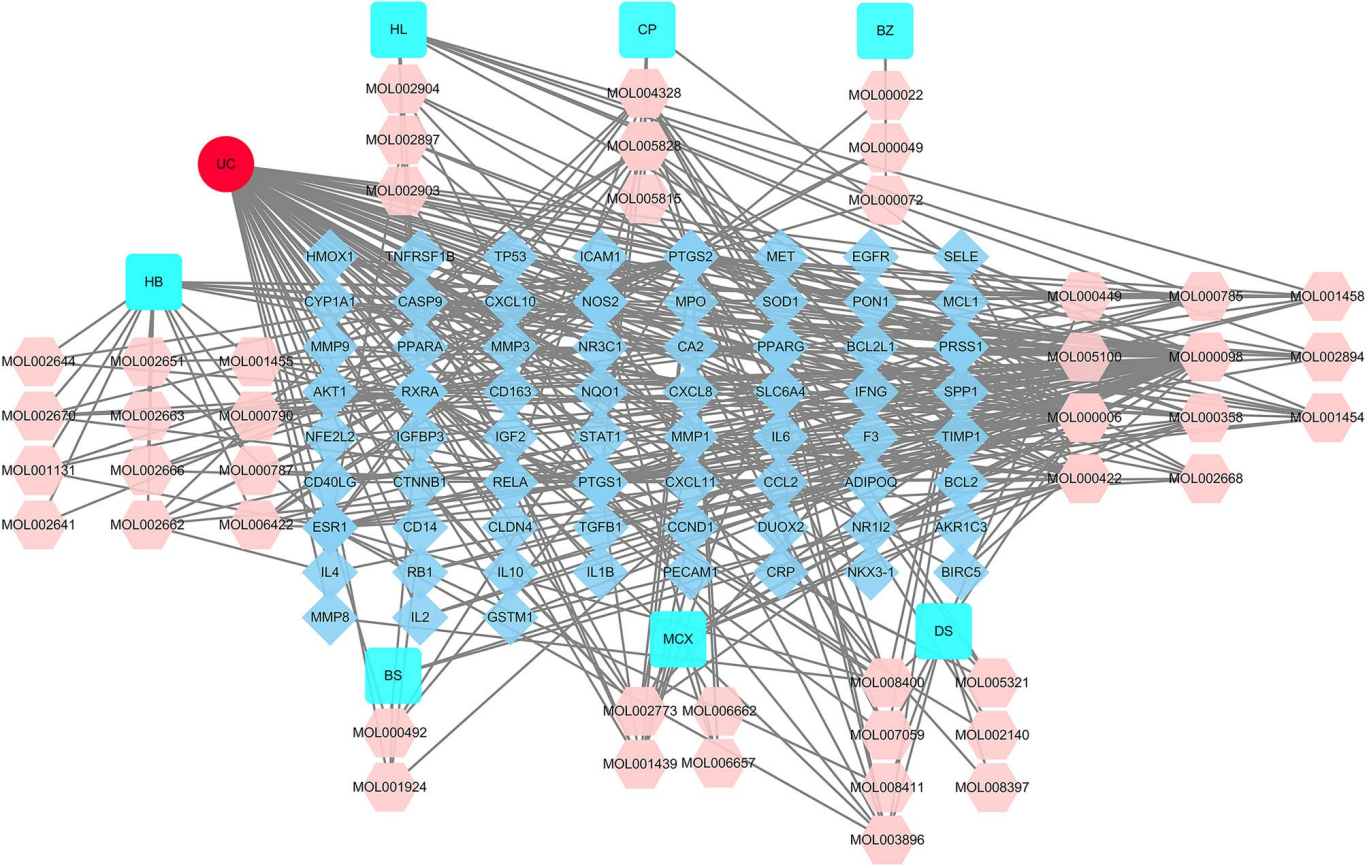
Common targets of SZLBD and UC. The red represents UC (ulcerative colitis), the teal represents traditional Chinese medicine (HL, Huanglian; CP, Chenpi; BZ, Baizhu; HB, Huangbai; MCX, Machixian; BS, Baishao; DS, Dangshen), the pink represents active ingredients, and the blue represents the targets of SZLBD acting on UC.

According to GeneCards and NCBI databases, 547 UC-related genes were obtained. A total of 67 common targets of SZLBD-UC were obtained by intersecting SZLBD-related targets and UC-related targets. The UC-herb-ingredient-target network is displayed in Fig. 3.

### PPI network of SZLBD-UC

**Figure 4:**
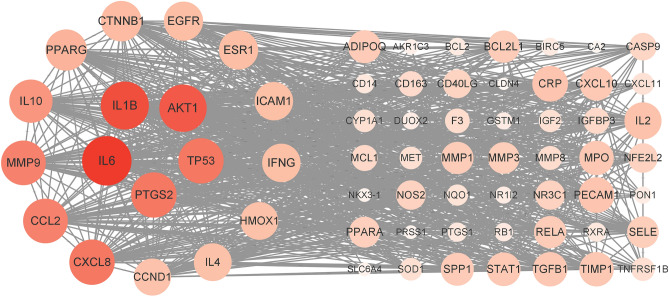
PPI network.

A PPI network with 67 nodes and 912 edges was constructed by using the STRING database and visualized through Cytoscape software (Fig. 4). Based on the topological analysis results of PPI network, we found that IL-6, IL-1B and AKT1 had high degree values, so we speculated that the above targets may be the key targets for SZLBD treatment of UC.

### GEO verification

**Figure 5:**
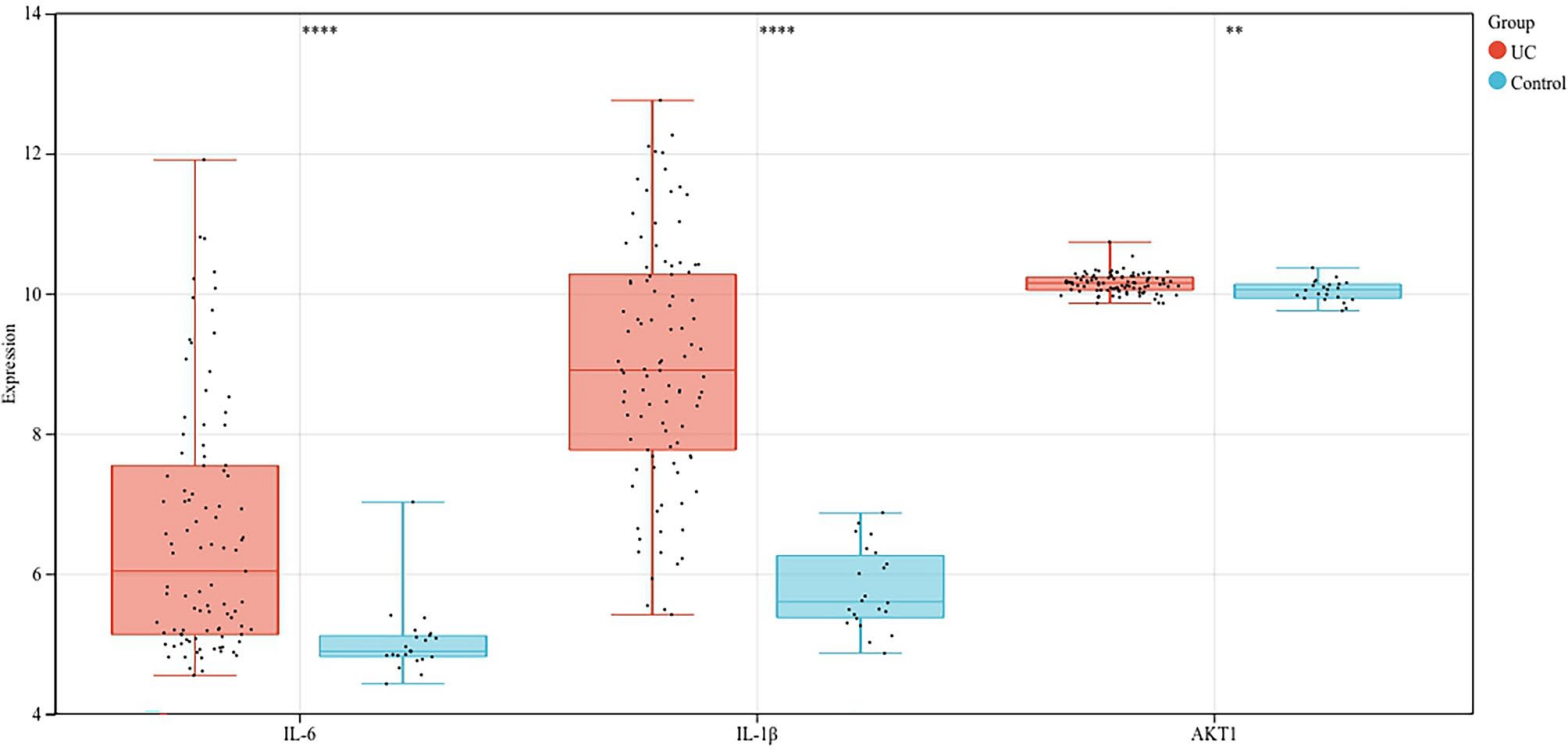
Expression of IL-6, IL-1B and AKT1 between normal and UC samples. **p < 0.01,****p < 0.001.

Based on the GSE75214 dataset (which includes 11 control samples and 97 UC patient samples), we analyzed the expression levels of IL-6, IL-1B, and AKT1 between the control and UC groups. The results showed that compared to the control group, the expression levels of IL-6, IL-1B, and AKT1 were higher in the UC group (Fig. 5).

### GO functional annotation and KEGG signaling pathway enrichment analysis

**Figure 6:**
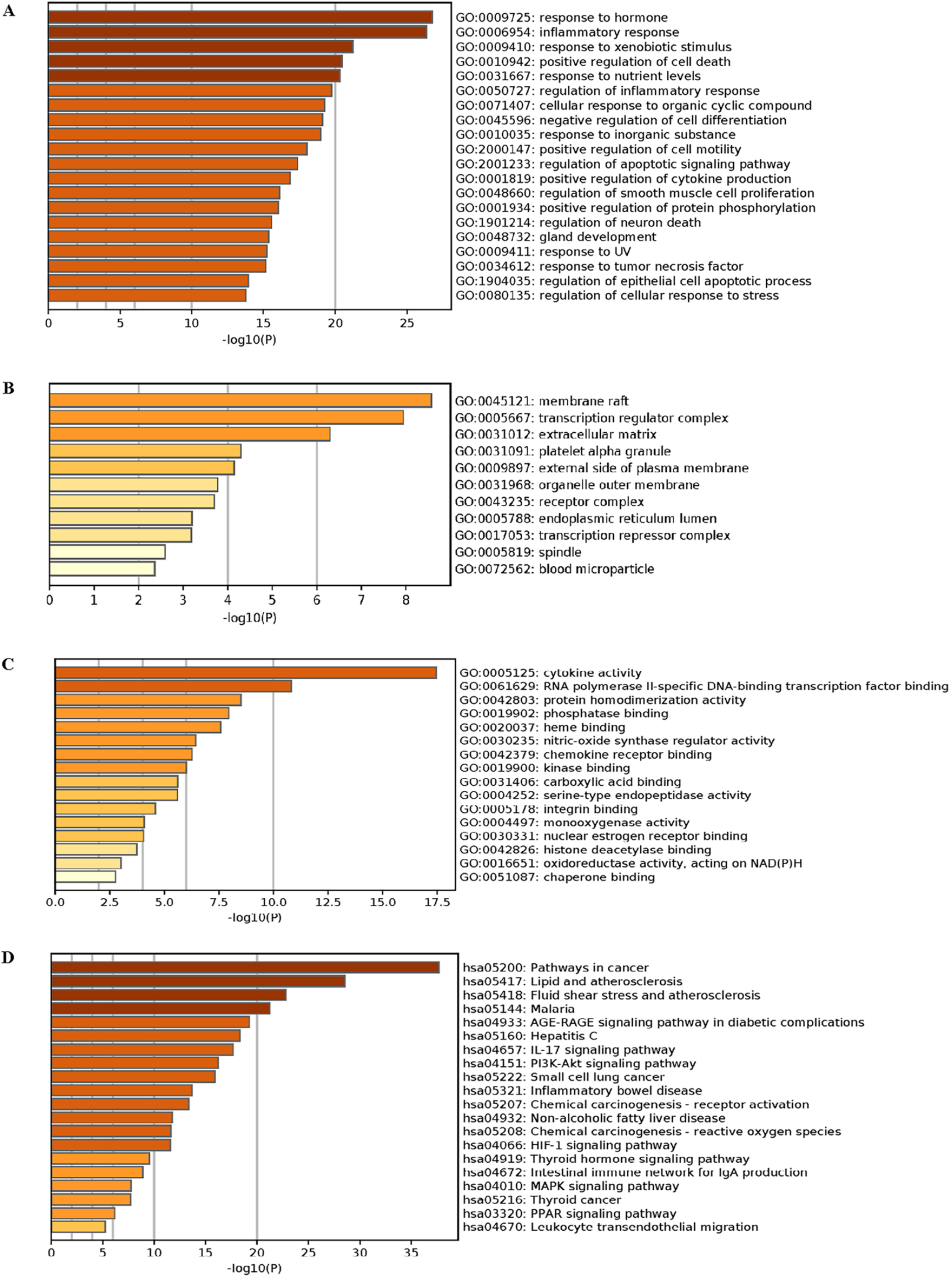
GO functional annotation and KEGG signaling pathway enrichment analysis results. (**A**) BP enrichment analysis; (**B**) CC enrichment analysis; (**C**) MF enrichment analysis (**D**) KEGG enrichment analysis.

A total of 1186 items were obtained from GO functional annotation analysis, and the significant enrichment results are shown in Fig. 6. Among them, there were 1072 BP entries (Fig. 6A), mainly involving inflammatory response, positive regulation of cell death and regulation of inflammatory response, etc. There were 34 CC entries (Fig. 6B), mainly involving membrane raft, transcription regulator complex and extracellular matrix, etc. There were 80 MF entries (Fig. 6C), involving cytokine activity, RNA polymerase II-specific DNA-binding transcription factor binding and protein homodimerization activity, etc.

A total of 138 pathways were obtained from KEGG signaling pathway enrichment analysis, and the significant results are shown in Fig. 5D. Signaling pathways associated with UC therapy mainly include IL-17 signaling pathway, PI3K-Akt signaling pathway and inflammatory bowel disease, etc.

### Molecular docking

**Figure 7:**
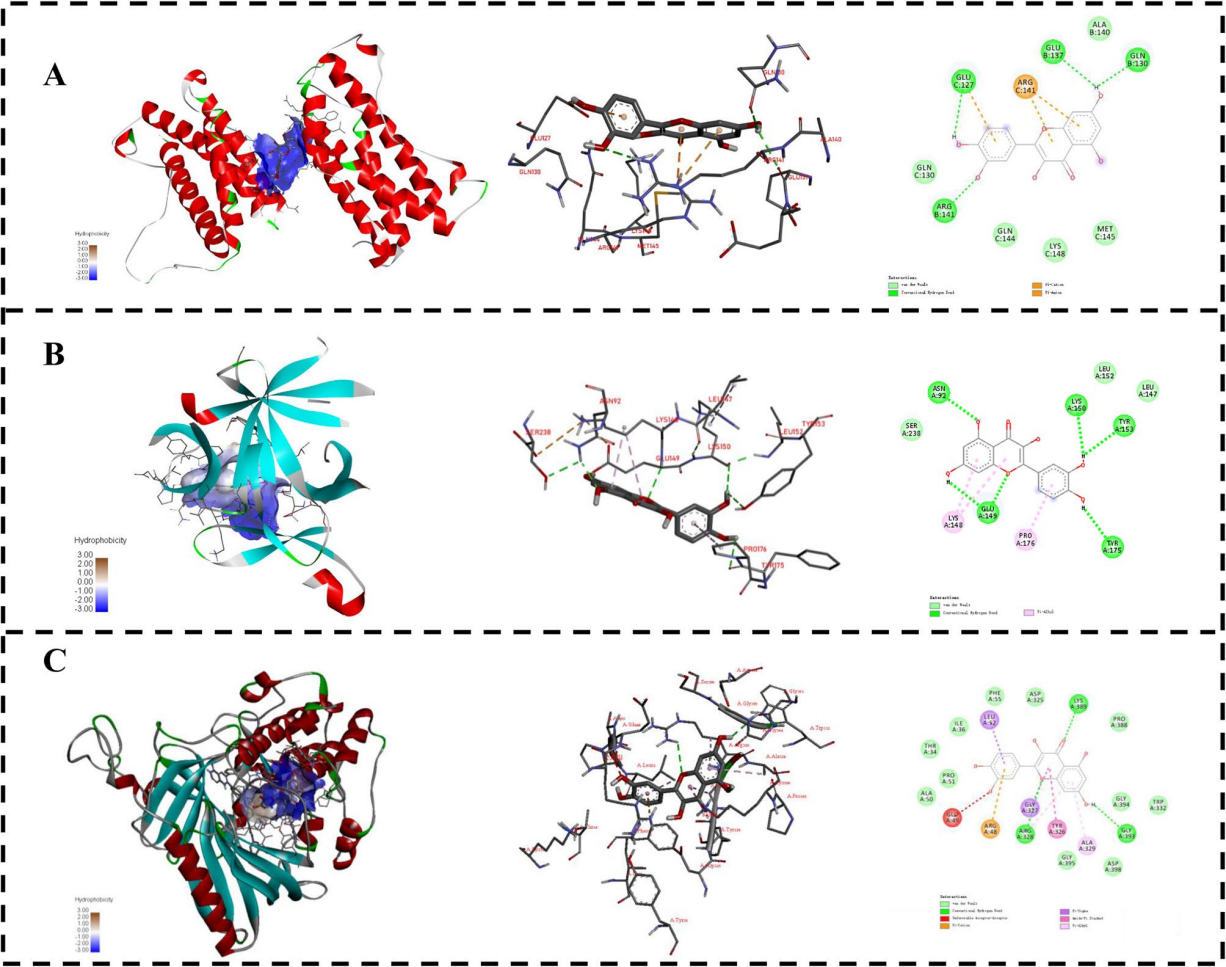
Molecular docking of quercetin (MOL000098) with its targets. (**A**) IL-6 protein-quercetin; (**B**) IL-1B protein-quercetin; (**C**) AKT1 protein-quercetin.

In this study, the main active ingredient quercetin was selected as the ligand molecule and the key targets IL-6, IL-1B and AKT1 were selected as receptor molecules for molecular docking, and the results showed that quercetin had a strong affinity with IL-6, IL-1B and AKT1 (Fig. 7). Quercetin and IL-6 formed stable complexes by interacting with amino acid GLU127, ARG141, GLU137 and GLN130 (Fig. 7A). Quercetin mainly interacted with amino acid residues ASN92, LYS148, CLU149, PRO176 and TYR175 of IL-1B (Fig. 7B). In addition, quercetin mainly interacted with AKT1 residues LEU52, GLU49, ARG48, CLY327, ARG328, TYR326, ALA329 and GLY393 (Fig. 7C). The molecular docking scores of quercetin with IL-6, IL-1B and AKT1 proteins were − 7.2 kcal/mol, − 7.0 kcal/mol and − 9.2 kcal/mol, respectively. Based on molecular docking, it was preliminarily verified that quercetin had good binding ability to IL-6, IL-1B and AKT1, which further proved the reliability of the prediction results of the network pharmacology.

### Experimental verification

**Figure 8:**
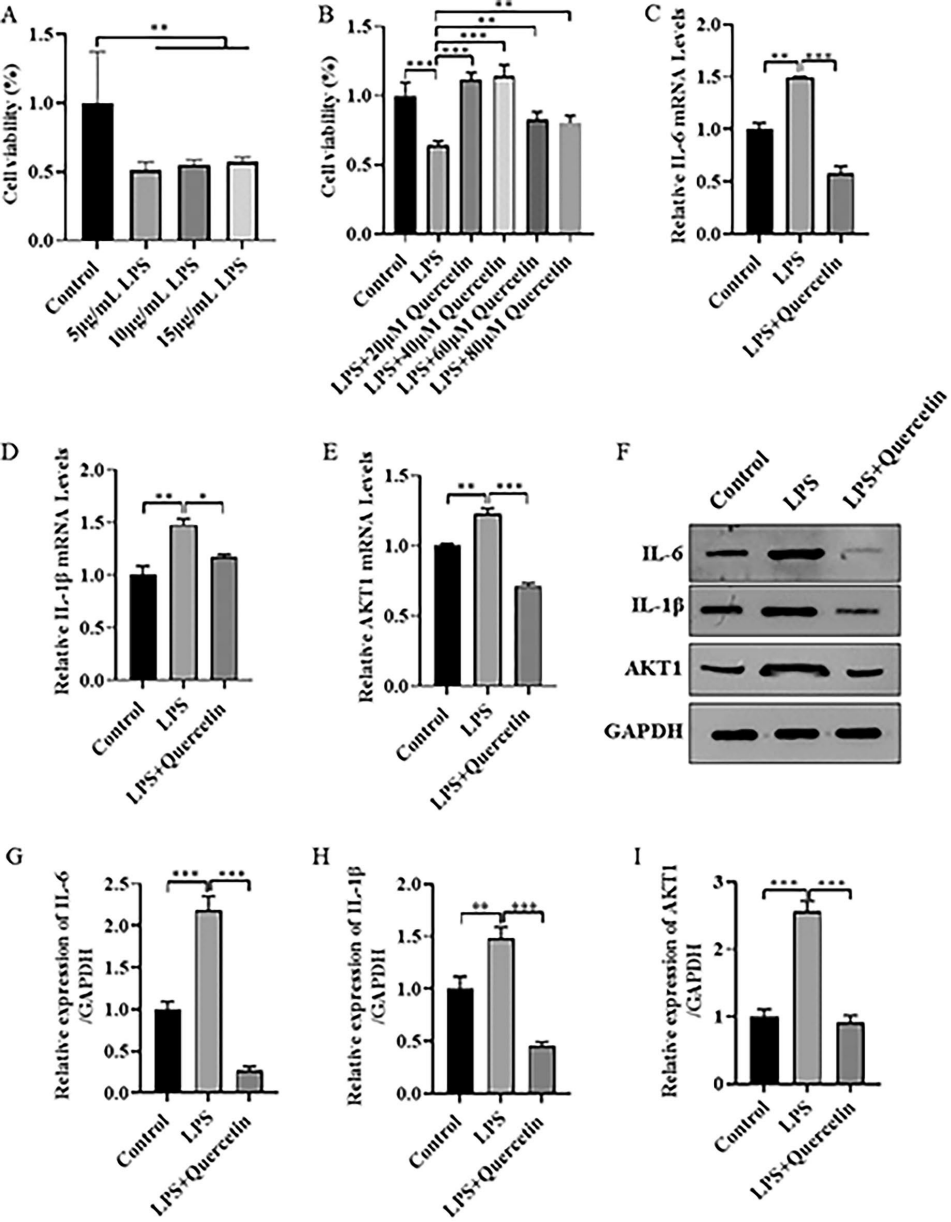
Quercetin improves LPS-induced inflammatory damage of HCoEpiC cells. (**A**) The choice of LPS safety and effective concentration; (**B**) The choice of quercetin safety and effective concentration; (**C**) Levels of mRNA expression of IL-6; (**D**) Levels of mRNA expression of IL-1B; (**E**) Levels of mRNA expression of AKT1; (**F**–**I**) Expression of IL-6, IL-1B and AKT1 proteins. *p < 0.05,**p < 0.01,***p < 0.001.

In order to verify that quercetin can interact with IL-6, IL-1B and AKT1 to participate in the treatment of UC by SZLBD, cellular validation was performed in this study. Firstly, an inflammatory cell model was constructed by treating with DMEM containing different concentrations of LPS (5, 10, 15 μg/mL) for 24 h. Cell viability was determined, and the optimal concentration was determined to be 10 μg/mL (Fig. 8A). After treating with LPS (10 μg/mL) for 24 h, cells were treated with different concentrations of quercetin (0, 20, 40, 60, and 80 μM) for 24 h, and cell viability was measured to determine the optimal concentration, which was found to be 40 μg/mL (Fig. 8B). We examined the effect of quercetin on inflammatory factors in HCoEpiC cells. Compared with the control group, the mRNA and protein expression levels of IL-6, IL-1B, and AKT1 were significantly increased in the model group. Compared with the model group, the mRNA and protein expression levels of IL-6, IL-1B, and AKT1were significantly decreased in the quercetin-treated group (Fig. 8C–I).

## Discussion

Literature has reported that Huanglian significantly inhibits the level of proinflammatory cytokines (IL-17) in the treatment of UC^15^. It has been found that Huangbai can improve chronic inflammatory injury caused by endothelial dysfunction by regulating the PI3K-Akt-mTOR signaling pathway^16^. Baizhu and Chenpi are the main ingredients of Baizhu Shaoyao San, another Chinese medicine for the treatment of UC, which can regulate inflammatory factors and intestinal flora^17^. Machixian has anti-ulcer and anti-inflammatory effects. Studies have shown that Machixian can reduce the symptoms of colitis^18^. Dangshen and Baishao are herbs from TCM formulas Zhen-Wu-Bu-Qi Decoction for the treatment of UC^19^, which exhibit anti-inflammatory properties by modulating the PI3K-AKT, MAPK signaling pathway, and NF-κB signaling pathway. In our study, 90 active compounds and 185 targets of SZLBD were identified from the TCMSP database. The compounds-targets network diagram shows the potential synergies between multiple ingredients and their targets. Quercetin, the main active ingredient, comes from Huanglian, Huangbai and Machixian*.* In conclusion, these three herbs of SZLBD can treat and improve UC by acting on the IL-17 and PI3K-Akt signaling pathways, which is consistent with and more complete than previous studies.

Quercetin (3, 5, 7, 3’, 4’-pentahydroxyflavonoid) is one of the main representatives of flavonoids. In plants, it exists mainly in the form of glycosides. In addition, quercetin has a wide range of biological effects, including antioxidant, anticancer, anti-inflammatory, antidiabetic and antibacterial effects^20^. It exerts its anti-inflammatory effect mainly by inhibiting the release of cytokines, reducing the production of COX and LOX, and maintaining the stability of mast cells^21^. A study of pulpitis has reported that quercetin reduces the production of pro-inflammatory cytokines IL-6 and IL-17^22^. Besides, quercetin-induced miR-369-3p modulates inflammatory cascades in chronic inflammatory responses and increases IL-6 production^23^. Quercetin can also inhibit inflammation by regulating the PI3K-AKT signaling pathway, thus reducing oxidative stress damage in GES-1 cells^24^. In mice with DSS-induced colitis, quercetin can significantly improve moderate DSS- induced colitis and reduce intestinal inflammation^25^. Additionally, in the study of Huai Hua San for treating UC, quercetin has also been found to play an important role in exerting anti-inflammatory effects^26^. In our study, both the prediction of active compounds and cell verification experiments indicated that quercetin was a key ingredient of SZLBD and played a vital role in the treatment of UC by regulating inflammation-related processes.

It is well known that *IL-1B* and *IL-6* are pro-inflammatory factors. In a past study, administration of a water-soluble artemisinin analog (SM934) was found to improve UC by reducing DSS-induced mRNA and protein levels of IL-1 B and IL-6^27^. Protein kinase B (*AKT*) is a member of the human serine-threonine kinase and the AGC family of protein kinases. Samples of human patients with UC showed severe down regulation of AKT1 function, accompanied by decreased *AKT1* activity and increased inflammation^28^. More drugs for the treatment of UC work through the pathway where AKT1 is located. Dihydroartemisinin can alleviate DSS-induced colitis via the PI3K-AKT signaling pathway^29^. Huangqin decoction ameliorates DSS-induced colitis by inhibiting the Ras-PI3K-Akt-HIF-1α pathway^30^. Quercetin can alleviate inflammation by reducing the levels of TNF-α, IL-1β, IL-6, and IL-10, thus treating DSS-induced colitis in mice^31^. The results of network pharmacology analysis and cell experiments in this study showed that quercetin, the main ingredient of SZLBD, targeted IL-6, IL-1B and AKT1 in the treatment of UC. We speculated that quercetin might improve UC by reducing the expression of pro-inflammatory factors and the PI3K-Akt signaling pathway.

Overall, this study suggested that quercetin might be the main active ingredient in SZLBD. *IL-6*, *IL-1B*, and *AKT1* were potential therapeutic targets of SZLBD in the treatment of UC. In addition, the role of SZLBD in UC might be achieved by modulating the balance of cytokines in the immune system and inflammation-related pathways, such as the IL-17 and PI3K-AKT pathways. These findings confirm that network pharmacology is an important method for predicting active compounds and targets. This study provides a theoretical basis for SZLBD in the treatment of UC and a comprehensive approach for finding more Chinese medicines to treat UC.

However, our study still has some limitations. First, most targets in the databases are verified, and unverified or undocumented ones may be overlooked. Second, quercetin, although identified as the main active ingredient of SZLBD, cannot completely replace SZLBD. Third, network pharmacology analysis ignores the absorption pathways, effective sites, active compounds, and metabolic forms of bioactive substances. Additionally, we have not yet explored the roles of other key ingredients within the decoction. Finally, we have not yet conducted animal studies to validate the efficacy of the SZLBD. Therefore, we need more in vitro and in vivo research to further explore the underlying molecular mechanisms by which SZLBD treats UC.

## Conclusion

In this study, we combined network pharmacology, molecular docking, and in vitro experiments to explore and verify the mechanism by which SZLBD targets and their main bioactive constituents effectively exert anti-UC effects. We found that SZLBD acted on multiple targets of multiple signaling pathways, and intervened in the PI3K-Akt and IL-17 pathways to delay the progression in UC. In the follow-up study, we plan to design in vivo and in vitro animal pharmacological experiments to deeply investigate the mechanism of action of SZLBD in the treatment of UC, so as to provide more references for its clinical application and development.

## Data Availability

All data generated or analysed during this study are included in this article. Further inquiries can be directed to the corresponding authors.

## Abbreviations

UC: Ulcerative colitis
SZLBD: Shen-Zhu-Lian-Bai decoction
TCM: Traditional Chinese medicines
TCMSP: Traditional Chinese Medicine Systems Pharmacology
PPI: Protein–protein interaction
BP: Biological process
CC: Cellular ingredient
MF: Molecular function
